# Histological image data of limb skeletal tissue from larval and adult Ambystoma mexicanum

**DOI:** 10.1016/j.dib.2016.07.028

**Published:** 2016-07-20

**Authors:** Catherine D. McCusker, Carlos Diaz-Castillo, Julian Sosnik, Anne Phan, David M. Gardiner

**Affiliations:** a Department of Biology, University of Massachusetts Boston, MA 02125, USA; b Department of Developmental and Cell Biology, University of California at Irvine, CA 92602, USA; c Department of Interdisciplinary Engineering, Wentworth Institute of Technology, Boston, MA 02115, USA; d Department of Cellular and Molecular Medicine, University of California San Diego, CA 92093, USA

**Keywords:** Cartilage, Bone, Periosteum, Limb, Ambystoma mexicanum

## Abstract

The data presented in this article are related to the article entitled “Cartilage and bone cells do not participate in skeletal regeneration in Ambystoma mexicanum limbs” [1]. Here we present image data of the post-embryonic development of the forelimb skeletal tissue of Ambystoma Mexicanum. Histological staining was performed on sections from the intact limbs of young (6.5 cm) and old (25 cm) animals, and on dissected skeletal tissues (cartilage, bone, and periosteum) from these animals.

**Specifications Table**

**Table:** 

Subject area	*Biology,*
More specific subject area	*Post-embryonic development*
Type of data	*Figure*
How data was acquired	*Leica Leitz microscope, and a Qimaging QIClick-F-M-12 camera controlled by Qimaging QCapture 2.9.13 software*
Data format	*Processed*
Experimental factors	*Histological analysis of intact or harvested limb skeletal tissue of 6.5 cm and 25 cm long Ambystoma mexicanum.*
Experimental features	*Analysis of Eosin, Hematoxylin, and Alician-blue-stained tissue sections*
Data source location	*Irvine, CA, United states*
Data accessibility	*Data is with this article*

**Value of the data**

• This data can be used to characterize the post-embryonic morphological changes in the limb skeletal tissue of larval and adult *Ambystoma mexicanum*.
• This data can be used to characterize the purity of dissected bone, cartilage, and periosteal tissues from the limb.
• The identification of contaminate perichondrium cells in the dissected cartilage tissue may help orient researchers to the challenges of manual purification.

## 1 Data

**Fig. 1:**
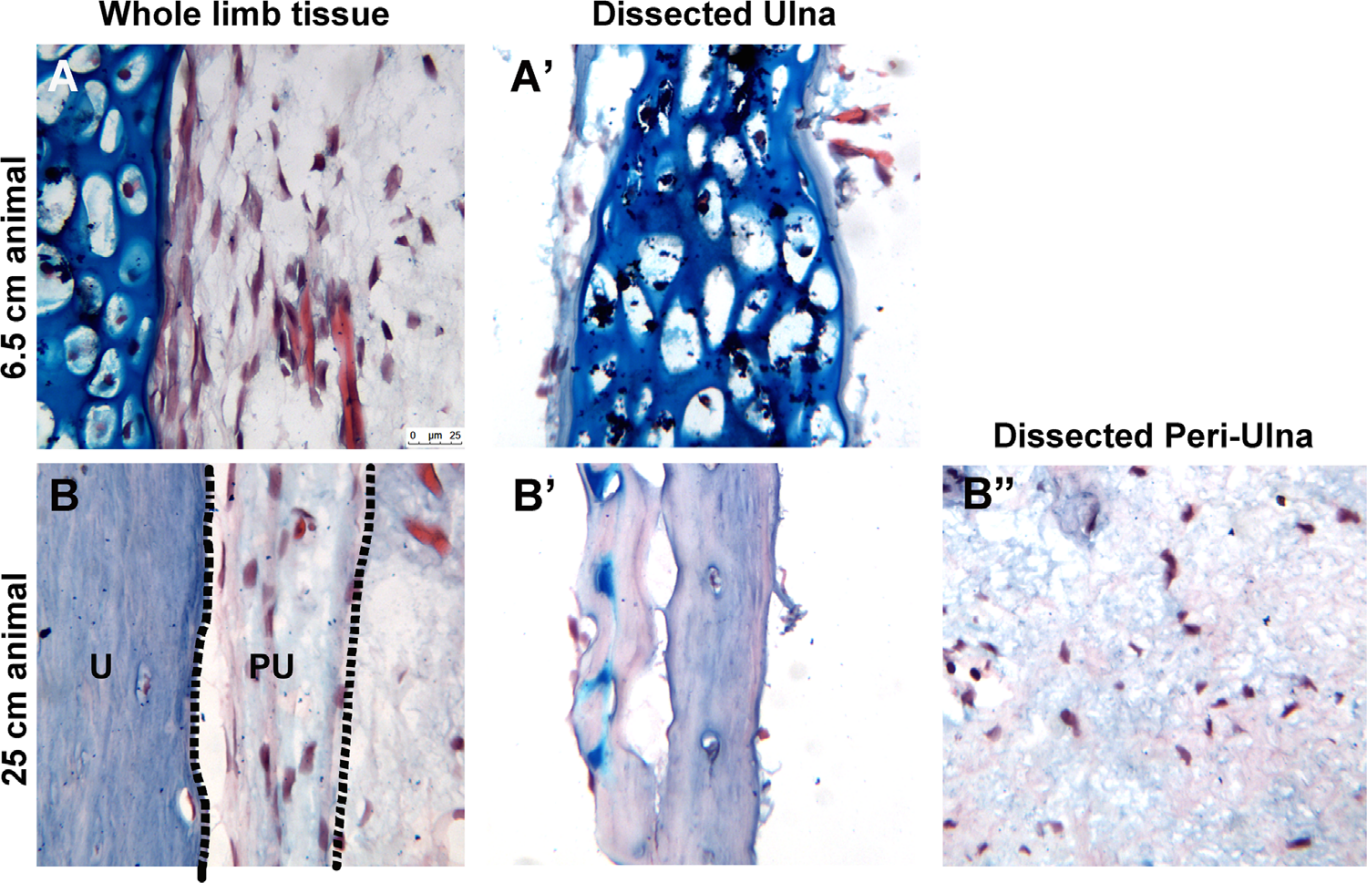
**Images of histology stained tissue sections of intact and dissected*****Ambystoma mexicanum*****limbs.** Bright field images were obtained of Eosin, Hematoxylin, and Alcian blue-stained longitudinal sections of intact (A, B) or dissected (A’, B’, B”) ulna tissue from “young” (6.5 cm length) and “old” (25 cm length) animals. Black dotted lines were drawn on the image of the whole limb tissue image from the 25 cm animal to delineate the boundary of the peri-ulna from the surrounding tissue (B). (A’ and B’) Dissected ulna tissue from 6.5 and 25 cm sized animals, respectively. (B”) Dissected peri-ulna tissue from 25 cm animals. Note the dissected ulna tissue from the 6.5 cm animal has contaminate (non-cartilage) tissue present. PU=peri-ulna, and U=ulna. The scale bar on “A” is 25 uM in length and is applicable for images A–B”.

Here we provide histological data of tissue sections from the limb skeletal tissues of larval (6.5 cm) and adult (25 cm) *Ambystoma mexicanum* (Fig. 1). The images exhibit the morphological characteristics of the limb skeletal tissue in the aging axolotl (6.5 cm compared to 25 cm long animals), and the purity of the skeletal tissues that have been harvested by manual dissection.

## 2 Experimental design, materials and methods

### 2.1 Animal husbandry and surgeries

This data was gathered in accordance with the recommendations in the Guide for the Care and Use of Laboratory Animals of the National Institutes of Health. The experimental work was approved by the Institutional Animal Care and Use Committee of the University of California Irvine. Mexican axolotls (*Ambystoma mexicanum*) were either spawned at the University of California, Irvine or obtained from the Ambystoma Genetic Stock Center, University of Kentucky. For all surgeries, animals were anesthetized using a 0.1% solution of MS222 (Ethyl 3-aminobenzoate methanesulfonate salt, Sigma), pH 7.0. Intact and dissected forelimb tissue were harvested and prepared for sectioning as described in [2].

### 2.2 Tissue processing

Tissue samples were fixed in 4% PFA in 1xPBS, embedded in OCT compound (Tissue-Tek) and sectioned longitudinally into 10 uM sections for histology staining. After washing sections three times with 1×PBS, they were stained with Hematoxylin, Eosin Y, and Alcian Blue as described in [2]. Color images were obtained of the intact or harvested ulna tissues using a Leica PL FLUOTAR 40×/0.70 objective mounted on a Leica Leitz DMRB Fluorescence microscope, and a Qimaging QIClick-F-M-12 camera controlled by Qimaging QCapture 2.9.13 software.
