# Supplementary material for: Histological image data of limb skeletal tissue from larval and adult Ambystoma mexicanum
Source: Data Brief. 2016 Jul 20;8:1206–8. doi: 10.1016/j.dib.2016.07.028 (PMC4982924; doi:10.1016/j.dib.2016.07.028)
Supplement: Supplementary file 1 — Supplementary material [file mmc1.docx]

**Conflict of interest form:**

The authors declare no conflict of interest in publishing this Data in Brief.

Catherine McCusker (on behalf of all authors) 6. 20.2016
